# Retinal Vascular Response to Hyperoxia in Patients with Diabetes Mellitus without Diabetic Retinopathy

**DOI:** 10.1155/2021/9877205

**Published:** 2021-10-15

**Authors:** Hamid Safi, Ramin Nourinia, Sare Safi, Ehsan Hadian, Bahareh Kheiri, Hamid Ahmadieh

**Affiliations:** ^1^Ophthalmic Research Center, Research Institute for Ophthalmology and Vision Science, Shahid Beheshti University of Medical Sciences, Tehran, Iran; ^2^Ophthalmic Epidemiology Research Center, Research Institute for Ophthalmology and Vision Science, Shahid Beheshti University of Medical Sciences, Tehran, Iran

## Abstract

**Purpose:**

To evaluate the retinal vascular response to hyperoxia in patients with diabetes at the preclinical stage of diabetic retinopathy (DR) and to quantify the changes in comparison with normal subjects using optical coherence tomography angiography (OCTA).

**Methods:**

In this prospective study, 40 eyes of 20 participants comprising 10 diabetic patients with no diabetic retinopathy (NDR) and 10 normal subjects were recruited. OCTA images were acquired in the resting position and were repeated after a hyperoxic challenge using a nasal mask connected to a reservoir bag supplying 100% oxygen at the rate of 15 L per minute for 5 minutes. The changes of mean parafoveal vessel density (VD) in the superficial capillary plexus (SCP) and deep capillary plexus (DCP), the foveal avascular zone (FAZ) size, and the outer retina flow index were compared between two conditions in each group and between the two study groups. The statistical significance of differences in the means was evaluated using Student's *t*-test for unpaired samples with consideration of the generalized estimating equations (GEE) for intereye correlation.

**Results:**

At baseline, the mean parafoveal VD of SCP and DCP were significantly lower in the NDR participants compared to the healthy subjects (*P* < 0.001 and *P* = 0.006, respectively). After induction of the hyperoxic challenge in healthy participants, mean parafoveal VD reduced at both the SCP and DCP, but reached a statistical significance only in DCP (*P* = 0.006). However, following induction of hyperoxic challenge in patients with NDR, no significant decline was noticed in mean parafoveal VD of SCP and DCP. The degree of change in mean parafoveal VD of DCP was statistically significantly more pronounced in healthy subjects compared to the NDR group (*P* = 0.034). The change in FAZ size and the outer retina flow index were comparable between the two study groups.

**Conclusion:**

Retinal capillary layers responded differently to hyperoxia-induced challenge, and in normal subjects, the autoregulatory mechanism was mostly effective in the parafoveal DCP. Retinal vascular reactivity was impaired in SCP and DCP at the preclinical stage of DR. OCTA as a noninvasive modality was able to quantify the retinal vascular response to the hyperoxic challenge.

## 1. Introduction

Retina exhibits the highest metabolic demand among other tissues in human [1]. Retinal blood flow is tightly regulated via the complex physiology of the vascular supply. The retinal vessels encompass the intrinsic ability to change their reactivity in response to alterations in hemodynamics, blood gas saturation, and intraocular pressure [2]. Evaluation of the autoregulatory mechanism would be possible by assessing the dynamic retinal vessel response to the excitatory stimuli. In particular, hyperoxic challenge in normal subjects can result in nearly one-third decline of retinal blood flow [3]. Alterations in retinal oxygen supply are believed to play an important role in various retinal vascular diseases including diabetic retinopathy (DR) [4]. The ability to directly observe retinal vasculature has been investigated using different retinal imaging technologies [5]. Although vascular reactivity has long been evaluated, its clinical acceptance is still a matter of debate because of low accuracy and low repeatability of various diagnostic instruments [6]. Optical coherence tomography angiography (OCTA) provides a noninvasive 3-dimensional imaginary of the retinal microvasculature and ability to measure the retinal blood flow at different posterior segment layers using novel software OCTA algorithms.

DR is the leading cause of visual impairment in the working age population worldwide. Currently, interventions are applied for DR once the irreversible microvascular damage has already happened [7]. Detection of retinal dysfunction at the preclinical stage can improve the management of DR [7]. OCTA may detect retinal microvascular changes in patients with diabetes mellitus (DM) without DR [8–10]. The attenuation of the retinal vascular autoregulatory mechanism has been established in patients with DR [11, 12]; however, there is a paucity of evidence for such changes at the preclinical stage of DR [13, 14]. OCTA has been utilized to demonstrate a significant change in the blood flow and vessel density (VD) of retinal peripapillary vasculature in response to hyperoxia challenge in healthy participants [6]. Sousa et al. utilized OCTA to assess the impairment of retinal vascular autoregulation during a hypoxia challenge in patients with type 1 DM with no diabetic retinopathy (NDR) [15]. However, the hyperoxia effect on the preclinical stage of DR in patients with type 2 DM has remained undetermined. Herein, we established a different setup to investigate the impairment of retinal vascular autoregulation secondary to hyperoxia in patients with type 2 DM and NDR. The purpose of this study was to compare retinal vessel reactivity in response to hyperoxia challenge between patients with DM without DR and healthy participants. The difference in the pattern of response would signify the potential ability of OCTA to show retinal autoregulatory dysfunction at the preclinical stage of DR.

## 2. Materials and Methods

This prospective study was conducted on 20 subjects (10 in the NDR group and 10 in the control group) referred to Labbafinezhad Medical Center, Tehran, Iran, between June 2020 and October 2020, and consisted of participants with type 2 DM without funduscopic signs of DR and age-matched normal subjects. The research protocol followed the tenets of the Declaration of Helsinki. The protocol of the study was approved by the Ethics Committee at the Ophthalmic Research Center, Shahid Beheshti University of Medical Sciences, Tehran, Iran. Details of the study were explained to participants, and a written informed consent was obtained. Subjects with significant media opacity, any associated macular pathology, history of smoking, history of glaucoma, history of systemic hypertension or cardiovascular disease, history of anemia, pulmonary disease and/or renal disease, any sign of active or history of ocular inflammation, and eyes with high refractive error were excluded. Subjects were asked to refrain from caffeine or tea for one day before the experiment. Axial length was measured using IOL Master500 (Carl Zeiss Meditec, Jena, Germany). Best corrected visual acuity was determined using the Snellen chart. Participants underwent standard ophthalmic examination including funduscopy with dilated pupils in order to reveal any sign of DR. Pulse rate and systolic and diastolic blood pressure of the participants were measured before and during the study.

The OCTA images (OCTA, RTVue XR Avanti, Optovue, USA, Version 2017.1.0.26) were acquired from both the eyes of the participants consecutively in resting and hyperoxic conditions. The software version included projection artifact removal (PAR) strategy in order to reduce artifacts in the DCP induced by the projection of superficial large retinal vessels. It allows the unmasking and proper visualization of DCP. OCTA of the macula was performed as a 6 × 6 mm angiographic image with the target fixated on the central fovea. The angio-analytics system provided by AngioVue software (AngioVue, RTVue XR Avanti; Optovue Inc.) was used to quantify the parameters. Parafoveal region was defined as a ring-shaped area between 1 and 3 mm diameters centered on the fovea. The procedure was repeated if the quality of the images were influenced by eye blinks, signal noise, or artifacts leading to a score lower than 7/10. The segment slabs of the superficial capillary plexus (SCP) and deep capillary plexus (DCP) enface image were automatically acquired. The segmentation was corrected manually by two masked independent readers (HS and MH) if the automated segmentation was inaccurate. For SCP, an inner boundary was set at 3 *μ*m beneath the internal limiting membrane and an outer boundary at 16 *μ*m beneath the inner plexiform layer. For DCP, an inner boundary was set at 16 *μ*m beneath the inner plexiform layer and an outer boundary at 72 *μ*m beneath the inner plexiform layer. The foveal avascular zone (FAZ) size was manually measured in square millimeters (mm^2^) and outlined by two masked independent readers (HS and MH). The size of the outlined FAZ area was calculated automatically by the OCTA software. Vessel density of the parafoveal regions were automatically calculated by dividing the number of pixels with flow signal by the total number of pixels multiplied by 100%. The flow index of the outer retina was defined as the average decorrelation value output in the 3.142 mm^2^ circular area centered in the foveal center of the enface retinal angiogram.

**Figure 1:**
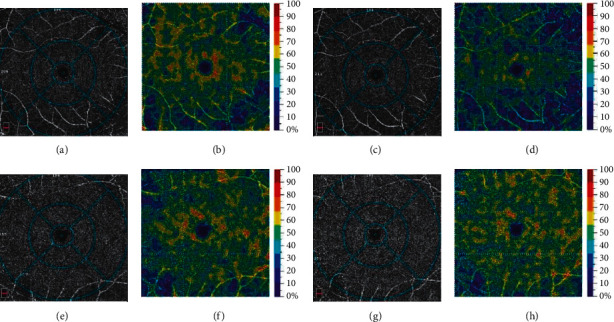
(a)–(d) Retinal angiogram and color-coded map of the right eye of the healthy participant at DCP before and after induction of hyperoxia. Vessel density at DCP significantly decreased in the parafoveal region after hyperoxic challenge. (e)–(h) Retinal angiogram and color-coded map of the left eye of the patient with diabetes mellitus without retinopathy at DCP before and after induction of hyperoxia. Vessel density at DCP showed minimal change in the parafoveal region after hyperoxic challenge. DCP, deep capillary plexus.

Participants were asked to rest in the sitting position taking a normal breath. Baseline OCTA images from both the eyes were recorded at the end of 10 minutes rest break. The hyperoxia protocol consisted of hyperventilation using a nasal mask connected to a 100% oxygen reservoir at the rate of 15 L per minute for 5 minutes. The second OCTA image was acquired at the end of the hyperventilation period (Figure 1). As the influence of hyperoxic status on retinal blood flow sustains for several minutes (2–4 minutes) [16], we acquired the OCTA image just after cessation of the hyperoxic challenge in the first eye followed by immediate imaging of the second eye. The overall time of imaging was under 2 minutes.

The statistical analysis was performed using a commercially available statistical software program (SPSS for Mac, version 25.0; IBM/SPSS, Chicago, IL, USA). The mean, range, and standard deviations of OCTA image indices at parafoveal SCP and DCP were calculated to present data. The OCTA parameters before and after hyperoxic challenge in both groups were compared using the paired *t*-test. The statistical significance of differences in the means between the NDR group and the control group was evaluated using Student's *t*-test for unpaired samples. We used GEE (generalized estimating equations) analysis to evaluate the possible intereye correlation. To calculate the power of the study, we performed a post hoc analysis. In regards to the lack of well-defined clinically significant differences in terms of the studied parameters, we considered figures of 5 and 10% of the original values as significant differences. *P* value less than or equal to 0.05 was considered statistically significant.

## 3. Results

In this prospective study, 40 eyes of 20 participants comprising 10 diabetic patients with NDR and 10 normal subjects were enrolled. The mean age was 42 ± 8 and 44 ± 7 years in NDR and control groups, respectively. Of these, 12 (60%) participants were female. The distribution of age and sex was comparable between the two groups. The mean duration of DM at the time of diagnosis was 8.60 ± 3.31 years (range: 2–14 years) in patients with NDR. During the study protocol, no participant faced any unstable hemodynamic condition. Demographic data are given in Table 1.

The mean baseline VD at the parafoveal SCP and DCP was statistically significantly lower in the NDR group compared to the healthy subjects (*P* < 0.001 and *P* = 0.006, respectively). However, the size of the FAZ and the flow index of the outer retinal layers were comparable between the two groups.

Following the hyperoxic challenge in healthy subjects, the parafoveal VD at both SCP and DCP decreased; however, a significant reduction was noticed only in the parafoveal DCP (*P* = 0.006). The FAZ area did not change in size. In NDR patients undergoing hyperoxic challenge, the mean parafoveal VD at SCP and DCP did not reduce compared to the baseline condition. The comparative change of VD after inducing the hyperventilation between the NDR and the control groups was statistically significant in terms of the parafoveal DCP parameters (Figure 1). The flow index in the outer retina declined from 1.204 ± 0.365 to 1.155 ± 0.423 and from 1.338 ± 0.623 to 1.150 ± 0.583 at baseline and after hyperoxic challenge in patients with NDR and in control subjects, respectively (*P* = 0.28). The FAZ area did not change in size (Table 2).

The study was adequately powered to detect the meaningful differences for changes in VD at DCP. The power for parafoveal VD at SCP, FAZ, and flow index (outer retina) to detect 5% and 10% clinically significant differences were 44% and 95%, 8% and 18%, and 7% and 14%, respectively.

## 4. Discussion

The autoregulatory mechanism is disturbed in patients with DM [10, 12] even at the preclinical stage of DR because of early neurovascular damage [17]. The impairment was documented at the early preclinical stage of DR using flicker stimulation [18, 19]. In addition, supernormal retinal oxygenation response was detected by magnetic resonance imaging retinal oximetry in patients with type I DM without clinically detectable retinopathy [20]. The mechanism of the effect of DM on vascular response to hyperoxia is not fully recognized. Besides pericyte destruction, DM causes loss of retinal vascular smooth muscle cells that regulate retinal vascular tone [21]. Moreover, responses to several vasogenic factors that influence retinal arteriolar tone and blood flow may be altered by DM [22].

At present, there is a lack of a noninvasive diagnostic method with sufficient reliability and clinical acceptance for the preclinical stage of DR [7]. Retinal vascular autoregulation disturbance may be a useful diagnostic indicator for the onset of DR. Sousa et al. utilized OCTA to demonstrate an impaired retinal vascular response during hypoxic challenge in patients with NDR [15]. We also reached the same conclusion. In contrast to normal subjects, we did not find significant changes in the parafoveal VD of neither the SCP nor the DCP after hyperoxic challenge in the NDR group. In contrast to the Sousa study, our results revealed that the macular capillary plexus layers had significantly different susceptibility to the extrinsic stimuli in healthy participants. In response to the hyperoxic status, the parafoveal VD of the DCP was the most affected parameter. Another difference between our study and the study by Sousa et al. was the type of DM. In the study by Sousa et al., only patients with type 1 DM were recruited. The trend of disease progression at the preclinical stage of DR is different between the two types of DM. In a recent meta-analysis, Zhang et al. elucidated that microvascular impairment in OCTA of subjects with NDR were more noticeable in type 2 DM [23]. It is believed that patients with type 1 DM tend to experience a quiescent period followed by a rapid progression to the nonproliferative stage. Thus, it may result in a wider variation of the severity of type 1 DM microvascular impairment at the preclinical stage. The third difference was the study protocol. The response model and response time of retinal blood flow alteration to the changes in the amount of oxygen saturation were different between the hypoxic and hyperoxic states [24, 25]. It might reflect the different compensatory mechanisms to regulate the oxygen concentration [26].

This prospective study revealed parafoveal VD reduction in the SCP and DCP in patients with NDR compared to healthy subjects. It reflects early vascular damage at the preclinical stage of DR that may ultimately lead to retinal capillaries dropout [7]. We noticed no significant difference in FAZ; this may be explained by the high interindividual variability of the FAZ size [27, 28]. A recent meta-analysis of OCTA findings in NDR patients demonstrated an enlarged FAZ area and lower VD in the macular SCP and DCP compared to healthy subjects [23]. Simonett and coworkers noticed significant reduction of the VD in the parafoveal DCP along with the unchanged FAZ area in patients with type 1 DM and NDR compared to healthy controls [29]. Note that the result of the current study in regards of FAZ was affected by the small sample size. Overall, basal retinal blood flow is altered in DM and leads to retinal hypoperfusion at the early stages of DR [30].

We acquired OCTA image with an OCTA software version including the PAR algorithm which differentiated from the traditional projection artifact removal algorithm by adding data from intensity gradient along the *Z*-axis and suppression of the projection artifacts to the level of the background noise. Therefore, PAR software maintains signal strength to better display the retinal vasculature. In particular, the vessel density of DCP was significantly greater using the PAR algorithm compared to the non-PAR algorithm at the NDR stage. Therefore, the results of previous reports measuring VD especially at DCP in DR with non-PAR software should be cautiously interpreted [31]. Hagag and his coworkers evaluated the effect of hyperoxia in 3 retinal capillary plexuses in healthy participants. Their results showed a significant reduction in VD only in DCP that was comparable to our findings [32]. Hyperoxic challenge causes high level of oxygen concentration in the outer retinal layers and photoreceptors because of the lack of autoregulatory mechanism in the choroid. Therefore, the higher compensatory response of DCP compared to SCP may be explained by the proximity of DCP to the region with oxygen supply from choroidal vasculature. Another reason may be the anatomical distribution of the capillary plexus of the inner retina. Superficial vascular plexus (SVP) consists of both large and small vessels with anastomosis to other vascular plexuses. In contrast, DCP is characterized by terminal anastomotic capillary networks nourished by anastomosis from SCP [33]. Consequently, parafoveal capillary nonperfusion initially occurs at DCP in patients with type 1 DM with no DR [28]. Hence, the autoregulatory response to hyperoxic challenge on DCP would be earlier and more pronounced compared to SCP.

There were a few limitations in the current study. The study might be influenced by the small sample size especially in interpretation of the results of FAZ and flow index parameters. However, the study was adequately powered regarding the main outcome measures (changes in VD at SCP and DCP). Although the study protocol for hyperoxic challenge was performed similarly for all participants, we did not directly measure the changes of blood oxygen saturation during OCTA image acquisition after the hyperoxic challenge. Furthermore, the lack of reduction in VD found in diabetic patients might be due to the fact that VD was already lower before hyperoxia, and thus, the potential for further reduction decreased. In addition, although we excluded low-quality OCTA images, signal noise and artifacts were inevitable that might interfere with the interpretation of the results, especially regarding the flow index parameter. The flow index presented in our study measured the average of absolute decorrelation signal. However, future studies using the novel variable interscan time analysis (VISTA) algorithm measuring the relative blood flow speed would evaluate the changes of flow more accurately [34].

## 5. Conclusions

This study demonstrated retinal vascular reactivity impairment at the preclinical stage of DR. Disturbance of the autoregulatory mechanism was significantly more pronounced in the parafoveal DCP. OCTA as a noninvasive modality was able to quantify the retinal vascular response to the hyperoxic challenge. Establishment of a precise method to assess retinal blood flow regulation in future may have clinical implications on evaluation of DR progression and management protocols.

## Figures and Tables

**Table 1:** Demographic characteristics of subjects.

	Groups			*P* value
	NDR	Control	Total	
Age, years				
Mean ± SD	42.20 ± 8.19	43.60 ± 7.40 7.407	42.9 ± 7.74	0.574
Range (min-max)	25 (29–54)	23 (32–55)	26 (29–55)	

SE, diopters				
Mean ± SD	+0.10 ± 0.50	+0.25 ± 0.51	+0.17 ± 0.50	0.350

AL, millimeters				
Mean ± SD	22.93 ± 0.78	23.24 ± 0.74	23.8 ± 0.76	0.205

Duration of DM, years				
Mean ± SD	8.60 ± 3.31	N/A	8.60 ± 3.31	N/A

Sex, *n* (%)				>0.999
Male	4 (40)	4 (40)	8 (40)
Female	6 (60)	6 (60)	12 (60)	

NDR, no diabetic retinopathy; DM, diabetes mellitus; min, minimum; max, maximum; SE, spherical equivalent; AL, axial length.

**Table 2:** Comparison of the OCTA parameters between NDR and control groups before and after the hyperoxic challenge.

	Groups		*P* value
	NDR	Control	
Parafoveal VD at SCP			
Before (mean ± SD)	44.64 ± 4.62	49.32 ± 3.95	<0.001
After (mean ± SD)	44.93 ± 6.44	48.75 ± 4.82	0.029
Difference	0.29 ± 3.41	−0.58 ± 4.96	0.51
*P* value	0.708	0.61	

Parafoveal VD at DCP			
Before (mean ± SD)	49.36 ± 5.32	53.06 ± 3.06	0.006
After (mean ± SD)	49.71 ± 5.42	50.17 ± 3.56	0.742
Difference	0.35 ± 6.75	−2.89 ± 4.19	0.034
*P* value	0.819	0.006	

FAZ (mm^2^)			
Before (mean ± SD)	0.285 ± 0.073	0.27 ± 0.087	0.536
After (mean ± SD)	0.332 ± 0.114	0.28 ± 0.083	0.093
Difference	0.05 ± 0.12	0.01 ± 0.03	0.173
*P* within	0.095	0.153	

Flow index, outer retina			
Before (mean ± SD)	1.338 ± 0.623	1.204 ± 0.368	0.396
After (mean ± SD)	1.15 ± 0.583	1.155 ± 0.423	0.972
Difference	−0.19 ± 0.37	−0.05 ± 0.47	0.281
*P* within	0.033	0.647	

Groups were compared using generalized estimating equation to correct for intereye associations. *P* value equal or less than 0.05 is significant and was calculated using the paired *t*-test. OCTA, optical coherence tomography angiography; NDR, no diabetic retinopathy; VD, vessel density; SCP, superficial capillary plexus; DCP, deep capillary plexus; FAZ, foveal avascular zone.

## Data Availability

The datasets generated during the current study are available from the corresponding author upon request.
